# Crystallization and X-ray diffraction analysis of an l-arabinonate dehydratase from *Rhizobium leguminosarum* bv. *trifolii* and a d-xylonate dehydratase from *Caulobacter crescentus*

**DOI:** 10.1107/S2053230X16010311

**Published:** 2016-07-13

**Authors:** Mohammad Mubinur Rahman, Martina Andberg, Anu Koivula, Juha Rouvinen, Nina Hakulinen

**Affiliations:** a Department of Chemistry, University of Eastern Finland, Joensuu Campus, PO Box 111, FIN-80101 Joensuu, Finland; b VTT Technical Research Centre of Finland Ltd, PO Box 1000, FIN-02044 VTT Espoo, Finland

**Keywords:** l-arabinonate dehydratase, d-xylonate dehydratase, IlvD/EDD enzymes, [Fe–S] cluster, *Rhizobium leguminosarum* bv. *trifolii*, *Caulobacter crescentus*

## Abstract

l-Arabinonate dehydratase (EC 4.2.1.25) and d-xylonate dehydratase (EC 4.2.1.82) are two enzymes that are involved in a nonphosphorylative oxidation pathway of pentose sugars. l-Arabinonate dehydratase converts l-arabinonate into 2-dehydro-3-deoxy-l-arabinonate, and d-xylonate dehydratase catalyzes the dehydration of d-xylonate to 2-dehydro-3-deoxy-d-xylonate. l-Arabinonate and d-xylonate dehydratases belong to the IlvD/EDD family, together with 6-phosphogluconate dehydratases and dihydroxyacid dehydratases. No crystal structure of any l-arabinonate or d-xylonate dehydratase is available in the PDB. In this study, recombinant l-arabinonate dehydratase from *Rhizobium leguminosarum* bv. *trifolii* (*Rl*ArDHT) and d-xylonate dehydratase from *Caulobacter crescentus* (*Cc*XyDHT) were heterologously expressed in *Escherichia coli* and purified by the use of affinity chromatography followed by gel-filtration chromatography. The purified proteins were crystallized using the hanging-drop vapour-diffusion method at 293 K. Crystals of *Rl*ArDHT that diffracted to 2.40 Å resolution were obtained using sodium formate as a precipitating agent. They belonged to space group *P*2_1_, with unit-cell parameters *a* = 106.07, *b* = 208.61, *c* = 147.09 Å, β = 90.43°. Eight *Rl*ArDHT molecules (two tetramers) in the asymmetric unit give a *V*_M_ value of 3.2 Å^3^ Da^−1^ and a solvent content of 62%. Crystals of *Cc*XyDHT that diffracted to 2.66 Å resolution were obtained using sodium formate and polyethylene glycol 3350. They belonged to space group *C*2, with unit-cell parameters *a* = 270.42, *b* = 236.13, *c* = 65.17 Å, β = 97.38°. Four *Cc*XyDHT molecules (a tetramer) in the asymmetric unit give a *V*_M_ value of 4.0 Å^3^ Da^−1^ and a solvent content of 69%.

## Abstract 2

l-Arabinonate dehydratase and d-xylonate dehydratase from the IlvD/EDD family were crystallized by the vapour-diffusion method. Diffraction data sets were collected to resolutions of 2.40 and 2.66 Å from crystals of l-arabinonate dehydratase and d-xylonate dehydratase, respectively.

## 1. Introduction   

**Figure 1:**
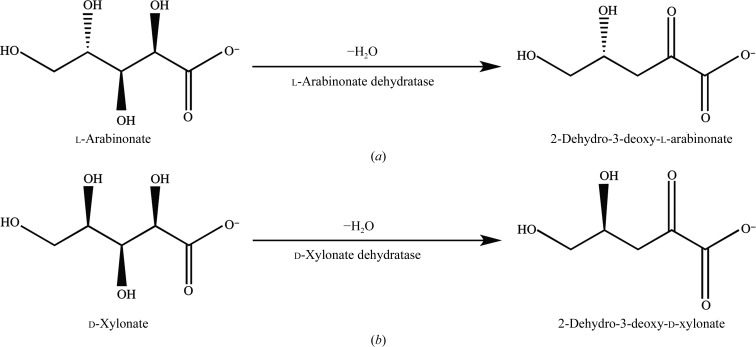
Catalytic reactions of (*a*) *Rl*ArDHT and (*b*) *Cc*XyDHT.

Lignocellulosic biomass is an important source of hexose and pentose sugars for the biorefinery industry. Apart from biofuels, the bioconversion of lignocellulosic hexose and pentose sugars can provide various platform chemicals which can be used as precursors for polymeric materials (Menon & Rao, 2012 ▸). The hemicellulose part of the lignocellulose biomass contains a significant amount of d-xylose and l-arabinose (Schädel *et al.*, 2010 ▸). In nature, some archaea, bacteria and fungi are known to metabolize l-arabinose (Boguta *et al.*, 2014 ▸; Brouns *et al.*, 2006 ▸; Dien *et al.*, 1996 ▸) and d-xylose (Okamoto *et al.*, 2014 ▸; Gu *et al.*, 2010 ▸) by using different nonphosphorylated oxidative pathways. In the Weimberg pathway, l-arabinose or d-xylose is converted to α-ketoglutarate, which is an intermediate metabolite in the tricarboxylic acid (TCA) cycle (Weimberg, 1961 ▸), or, in the Dahms pathway, the pentose sugars are converted to glycol­aldehyde and pyruvate (Dahms, 1974 ▸). In both pathways, l-arabinonate dehydratase (EC 4.2.1.25) catalyzes the removal of one water molecule from l-arabinonate to produce 2-dehydro-3-deoxy-l-arabinonate (Fig. 1 ▸ *a*). Similarly, d-xylonate dehydratase (EC 4.2.1.82) catalyzes the removal of one water molecule from d-xylonate to produce 2-dehydro-3-deoxy-d-xylonate (Fig. 1 ▸ *b*) (Stephens *et al.*, 2007 ▸; Dilworth *et al.*, 1986 ▸; Weimberg, 1961 ▸). Several bacterial and yeast strains have been engineered to metabolize l-arabinose or d-xylose through the oxidative pathway and to produce biofuels, in addition to other value-added industrial chemicals (Kurosawa *et al.*, 2015 ▸; Jin *et al.*, 2014 ▸; Demeke *et al.*, 2013 ▸; Xiao *et al.*, 2012 ▸; Bettiga *et al.*, 2009 ▸; Meijnen *et al.*, 2009 ▸; Matsushika *et al.*, 2009 ▸).

l-Arabinonate dehydratase from *Rhizobium leguminosarum* bv. *trifolii* and d-xylonate dehydratase from *Caulobacter crescentus* belong to the IlvD/EDD enzyme family. Enzymes in this family contain iron–sulfur [Fe–S] clusters as a prosthetic group (Andberg *et al.*, 2016 ▸; Nunn *et al.*, 2010 ▸; Stephens *et al.*, 2007 ▸) and, using spectroscopic methods, active enzymes from this family have been found to contain either a [4Fe–4S] cluster (Watanabe *et al.*, 2006 ▸; Rodriguez *et al.*, 1996 ▸; Flint *et al.*, 1993 ▸) or a [2Fe–2S] cluster (Flint & Emptage, 1988 ▸) in the active sites. There is only one crystal structure in the PDB from the IlvD/EDD family (PDB entry 2gp4; Southeast Collaboratory for Structural Genomics, unpublished work) and it lacks the [Fe–S] cluster. Therefore, structural studies of enzymes belonging to this family are needed in order to understand their structure–function relationship and the role of the [Fe–S] cluster. Here, we describe the overproduction, purification, crystallization and X-ray diffraction analysis of two enzymes from the IlvD/EDD family.

## 2. Materials and methods   

### 2.1. Macromolecule production   

The *araD* gene encoding *R. leguminosarum* l-arabinonate dehydratase (*Rl*ArDHT) and the *xylD* gene encoding *C. crescentus* d-xylonate dehydratase (*Cc*XyDHT) were purchased as codon-optimized synthetic genes from GeneArt, Germany and a *Strep*-Tag II (Trp-Ser-His-Pro-Gln-Phe-Glu-Lys) was added at the N-terminus (the deposited GenBank accession numbers for *araD* and *xylD* are KT260159 and KT260154, respectively). The genes were cloned into a pBAT4 expression vector, as described in Andberg *et al.* (2016 ▸). Other information on macromolecule production is given in Table 1 ▸.

For protein production, *Escherichia coli* BL21(DE3) expression cells were transformed with the recombinant plasmids. Large-scale protein production was performed in 2 l shake flasks containing 500 ml Luria–Bertani (LB) culture medium with 100 µg ml^−1^ ampicillin. The culture flasks were incubated at 303 K in a shaker incubator at 180 rev min^−1^. When the OD_600 nm_ reached 0.5–0.6, protein expression was induced by the addition of isopropyl β-d-1-thiogalactopyranoside (IPTG) to a final concentration of 1 m*M*, and cultivation was continued under identical conditions overnight (Andberg *et al.*, 2016 ▸). Cells were harvested from the culture medium by centrifugation at 4000 rev min^−1^ at 277 K for 25 min. The cell pellets were suspended in an extraction buffer consisting of 50 m*M* Tris–HCl pH 8.0, 150 m*M* NaCl, 5 m*M* MgCl_2_, 1 m*M* DTT, protease inhibitor (EDTA-free, Roche) and 100 µg ml^−1^ lysozyme (Sigma–Aldrich, Germany). Intracellular proteins were isolated by centrifugation at 18 500 rev min^−1^, followed by a one-step freeze–thawing of the suspended cells at 193 K and sonication.

Crude protein samples were loaded onto a StrepTrap HP 5 ml column (GE Healthcare, Sweden) equilibrated with binding buffer (50 m*M* Tris–HCl pH 8.0, 150 m*M* NaCl, 5 m*M* MgCl_2_). *Strep-*Tag II-bound proteins were eluted with elution buffer (50 m*M* Tris–HCl pH 8.0, 150 m*M* NaCl, 5 m*M* MgCl_2_, 2.5 m*M* d-desthiobiotin). A final polishing step was conducted by gel-filtration chromatography on a Superdex 200 HR 10/30 column (GE Healthcare, Sweden) equilibrated with a buffer consisting of 50 m*M* Tris–HCl pH 7.5, 5 m*M* MgCl_2_. Purified samples were concentrated using an Amicon Ultra-4 centrifugal filter device with a molecular-weight cutoff of 30 kDa (Merck Millipore, Germany). The molecular weights and the purity of the enzymes were checked by SDS–PAGE with silver staining in a PhastSystem (GE Healthcare, Sweden). The enzyme activities were checked by the thiobarbituric acid (TBA) assay and the semicarbazide assay (Andberg *et al.*, 2016 ▸).

### 2.2. Crystallization   

Crystallization of the purified enzymes was performed by the hanging-drop vapour-diffusion method in a 24-well cell-culture plate (Greiner Bio-One, Germany) at 293 K. The following commercial crystallization kits were used: Crystal Screen, Crystal Screen 2, PEG/Ion, SaltRx 1, SaltRx 2, Index HT, PEGRx 1 and PEGRx 2 (Hampton Research). Droplets were prepared by mixing protein sample and crystallization reagent in a 1:1 ratio on a siliconized glass cover slide. The cover slides were then turned drop-side down against 500 µl crystallization reservoir reagent and sealed with grease to ensure that they were airtight. The plates were stored at 293 K. A microseeding technique (Bergfors, 2003 ▸) was applied to improve the quality of the *Cc*XyDHT crystals. An initial condition that consisted of 3.6 *M* sodium formate, 5% PEG 3350 gave clusters of *Cc*XyDHT crystals. A small cluster of crystals was removed in 4 µl crystallization reagent on a glass cover slide, crushed with a micro needle, diluted with 46 µl crystallization reagent to a final volume of 50 µl and sonicated for 3 min in a sonication bath (FinnSonic, Finland). A dog hair was dipped into the seed-stock solution and streaked through pre-equilibrated crystallization drops. Crystallization information is shown in Table 2 ▸.

### 2.3. Data collection and processing   

*Rl*ArDHT crystals were mounted on a nylon loop and soaked in a cryoprotectant solution consisting of 4.0 *M* sodium formate, 0.1 *M* MES pH 7.0, 15% glycerol. *Cc*XyDHT crystals were soaked with 3.9 *M* sodium formate pH 7.0, 0.1 *M* TES pH 7.0, 5 m*M* magnesium formate, 4% PEG 3350, 40 m*M* calcium d-xylonate. Data collection for *Rl*ArDHT crystals was carried out at 100 K on beamline ID14-2 at the ESRF in France at a wavelength of 0.97957 Å. Data from *Cc*XyDHT crystals were collected remotely on beamline I04 at Diamond Light Source (DLS) in England at a wavelength of 0.97949 Å. Data processing was carried out by *XDS* (Kabsch, 2010 ▸). Detailed data-collection and processing statistics are shown in Table 3 ▸.

## 3. Results and discussion   

Purified dehydratase enzymes contain an [Fe–S] cluster, which is necessary for enzyme activity (Andberg *et al.*, 2016 ▸). Both the *Rl*ArDHT and the *Cc*XyDHT protein solutions were dark brown in colour, which was a good indication that the [Fe–S] clusters had been maintained. In addition, the purified proteins were found to be active by an enzyme-activity assay as described previously (Andberg *et al.*, 2016 ▸). Based on SDS–PAGE analysis (Supplementary Fig. S1), the molecular weights of the purified *Rl*ArDHT and *Cc*XyDHT were approximately 64 kDa, and are in good correspondence with the theoretical molecular weights. The calculated molecular weights are 63.738 and 64.314 kDa (without the [Fe–S] cluster) for *Rl*ArDHT and *Cc*XyDHT, respectively.

**Figure 2:**
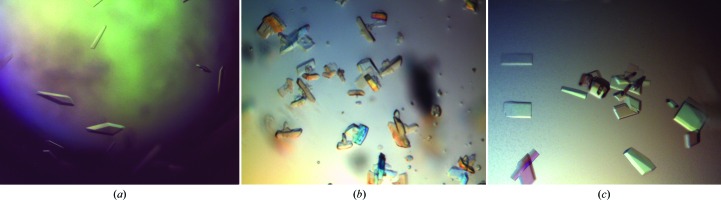
Crystals of (*a*) *Rl*ArDHT, (*b*) *Cc*XyDHT before seeding and (*c*) *Cc*XyDHT after seeding.

**Figure 3:**
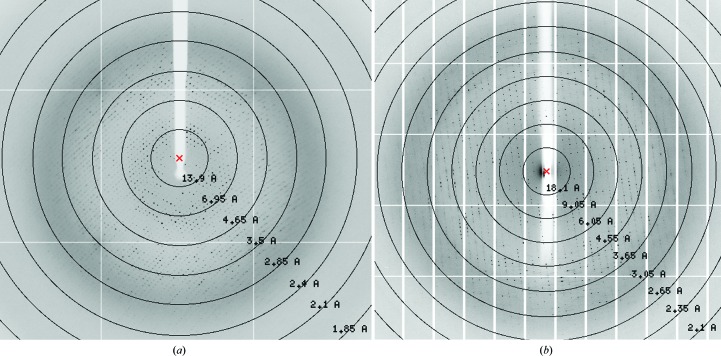
Diffraction images of (*a*) *Rl*ArDHT and (*b*) *Cc*XyDHT crystals. The black circles correspond to the resolution limit in Å.

*Rl*ArDHT was crystallized in 4.0 *M* sodium formate, 0.1 *M* MES pH 7.0, which was a modification of condition No. 33 (4 *M* sodium formate) from Crystal Screen. Crystals appeared within two weeks (Fig. 2 ▸ *a*) and diffracted to 2.4 Å resolution (Fig. 3 ▸ *a*). The space group was *P*2_1_, with unit-cell parameters *a* = 106.07, *b* = 208.61, *c* = 147.09 Å, β = 97.43°. Assuming eight molecules in the asymmetric unit, the calculated Matthews coefficient (*V*_M_) was 3.2 Å^3^ Da^−1^, which corresponds to a solvent content of 62% (Matthews, 1968 ▸).

In the initial crystallization screening of *Cc*XyDHT, hexagonal bipyramidal crystals were obtained in various conditions. Most of the conditions contained salts, while others contained PEGs as a precipitating agent. However, the crystals showed only poor diffraction to 6 Å resolution. Additive and detergent screens were also tried, but no real improvement was observed. A modified condition that consisted of 3.6 *M* sodium formate pH 7.0, 5% PEG 3350 gave a cluster of bar-shaped crystals. Microseeding, together with further modification of the crystallization condition (3.8 *M* sodium formate pH 7.0, 0.1 *M* TES pH 7.0, 5 m*M* magnesium formate, 4% PEG 3350) gave crystals within four weeks (Fig. 2 ▸ *b*) which diffracted to 2.66 Å resolution (Fig. 3 ▸ *b*). The crystals belonged to space group *C*2, with unit-cell parameters *a* = 270.42, *b* = 236.13, *c* = 65.17 Å, β = 97.38°. Assuming four molecules in the asymmetric unit, the calculated Matthews coefficient (*V*_M_) was 4.0 Å^3^ Da^−1^, which corresponds to a solvent content of 69% (Matthews, 1968 ▸).

The structures of *Rl*ArDHT and *Cc*XyDHT were both solved by molecular replacement using *Phaser* (McCoy *et al.*, 2007 ▸). The structure of *Rl*ArDHT was solved using a modified model from PDB entry 2gp4 as a template. This resulted in a clear solution with two tetramers (*Z*-score = 12) in the asymmetric unit, with initial *R* and *R*_free_ values of 37.7 and 46.7%, respectively. The structure of *Cc*XyDHT was subsequently solved by molecular replacement using the coordinates of *Rl*ArDHT. A clear solution with a tetramer in the asymmetric unit (*Z*-score = 9) was found and resulted in initial *R* and *R*_free_ values of 31.7 and 39.7%, respectively. Three-dimensional structure determination and structure refinement of both *Rl*ArDHT and *Cc*XyDHT are ongoing.

## Supplementary Material

Supplementary Figure S1.. DOI: 10.1107/S2053230X16010311/nj5256sup1.pdf

## Figures and Tables

**Table 1:** Macromolecule-production information

	*Rl*ArDHT	*Cc*XyDHT
Source organism	*R. leguminosarum* bv. *trifolii* (GenBank accession No. KT260159)	*C. crescentus* (GenBank accession No. KT260154)
DNA source	Synthetically made	Synthetically made
Forward primer†	CACGTCCATGGACTGGTCTCATCCACAATTCGAGAAGAAAAAAAAAGCTGAATGGCCG	ACGTCCATGGACTGGTCTCATCCACAATTCGAGAAGTCTAATCGCACCCCGCGTC
Reverse primer‡	GACGAATTCTCAATG	GTCGACGAATTCTCAATG
Cloning vector	pBAT4	pBAT4
Expression vector	pBAT4	pBAT4
Expression host	*E. coli* BL21(DE3)	*E. coli* BL21(DE3)
Complete amino-acid sequence of the construct produced§	MDWSHPQFEKKKKAEWPRKLRSQEWYGGTSRDVIYHRGWLKNQGYPHDLFDGRPVIGILNTWSDMTPCNGHLRELAEKVKAGVWEAGGFPLEVPVFSASENTFRPTAMMYRNLAALAVEEAIRGQPMDGCVLLVGCDKTTPSLLMGAASCDLPSIVVTGGPMLNGYFRGERVGSGTHLWKFSEMVKAGEMTQAEFLEAEASMSRSSGTCNTMGTASTMASMAEALGMALSGNAAIPGVDSRRKVMAQLTGRRIVQMVKDDLKPSEIMTKQAFENAIRTNAAIGGSTNAVIHLLAIAGRVGIDLSLDDWDRCGRDVPTIVNLMPSGKYLMEEFFYAGGLPVVLKRLGEAGLLHKDALTVSGETVWDEVKDVVNWNEDVILPAEKALTSSGGIVVLRGNLAPKGAVLKPSAASPHLLVHKGRAVVFEDIDDYKAKINDDNLDIDENCIMVMKNCGPKGYPGMAEVGNMGLPPKVLKKGILDMVRISDARMSGTAYGTVVLHTSPEAAVGGPLAVVKNGDMIELDVPNRRLHLDISDEELARRLAEWQPNHDLPTSGYAFLHQQHVEGADTGADLDFLKGCRGNAVGKDSH	MDWSHPQFEKSNRTPRRFRSRDWFDNPDHIDMTALYLERFMNYGITPEELRSGKPIIGIAQTGSDISPCNRIHLDLVQRVRDGIRDAGGIPMEFPVHPIFENCRRPTAALDRNLSYLGLVETLHGYPIDAVVLTTGCDKTTPAGIMAATTVNIPAIVLSGGPMLDGWHENELVGSGTVIWRSRRKLAAGEITEEEFIDRAASSAPSAGHCNTMGTASTMNAVAEALGLSLTGCAAIPAPYRERGQMAYKTGQRIVDLAYDDVKPLDILTKQAFENAIALVAAAGGSTNAQPHIVAMARHAGVEITADDWRAAYDIPLIVNMQPAGKYLGERFHRAGGAPAVLWELLQQGRLHGDVLTVTGKTMSENLQGRETSDREVIFPYHEPLAEKAGFLVLKGNLFDFAIMKSSVIGEEFRKRYLSQPGQEGVFEARAIVFDGSDDYHKRINDPALEIDERCILVIRGAGPIGWPGSAEVVNMQPPDHLLKKGIMSLPTLGDGRQSGTADSPSILNASPESAIGGGLSWLRTGDTIRIDLNTGRCDALVDEATIAARKQDGIPAVPATMTPWQEIYRAHASQLDTGGVLEFAVKYQDLAAKLPRHNH

† The NcoI site is underlined.

‡ The EcoRI site is underlined.

§ The *Strep*-Tag II is underlined.

**Table 2:** Crystallization

	*Rl*ArDHT	*Cc*XyDHT
Method	Hanging-drop vapour diffusion	Hanging-drop vapour diffusion
Plate type	24-well cell-culture plate	24-well cell-culture plate
Temperature (K)	293	293
Protein concentration (mg ml^−1^)	9.0	7.5
Buffer composition of protein solution	50 m*M* Tris–HCl pH 7.5, 5 m*M* MgCl_2_	50 m*M* Tris–HCl pH 7.5, 5 m*M* MgCl_2_
Composition of reservoir solution	4.0 *M* sodium formate, 0.1 *M* MES pH 7.0	3.8 *M* sodium formate pH 7.0, 0.1 *M* TES pH 7.0, 5 m*M* magnesium formate, 4% PEG 3350
Volume and ratio of drop	4 µl, 1:1	4 µl, 1:1
Volume of reservoir (µl)	500	500

**Table 3:** Data collection and processing Values in parentheses are for the outer shell.

	*Rl*ArDHT	*Cc*XyDHT
Diffraction source	ID14-2, ESRF, France	I04, DLS, England
Wavelength (Å)	0.97957	0.97949
Temperature (K)	100	100
Detector	CCD	Pilatus 6M
Crystal-to-detector distance (mm)	335.51	519.95
Rotation range per image (°)	0.25	0.5
Total rotation range (°)	180	180
Exposure time per image (s)	0.1	0.1
Space group	*P*2_1_	*C*2
*a*, *b*, *c* (Å)	106.07, 208.61, 147.09	270.42, 236.13, 65.17
α, β, γ (°)	90, 90.43, 90	90, 97.38, 90
Mosaicity (°)	0.65	0.7
Resolution range (Å)	40.00–2.40 (2.50–2.40)	59.69–2.66 (2.73–2.66)
Total No. of reflections	779327 (88849)	393801 (60419)
No. of unique reflections	246940 (28307)	114638 (17945)
Completeness (%)	99.3 (99.0)	99.0 (95.8)
Multiplicity	3.2 (3.1)	3.4 (3.4)
〈*I*/σ(*I*)〉	12.3 (2.0)	17.2 (2.1)
CC_1/2_	99.5 (57.8)	99.9 (75.1)
*R*_meas_ (%)	10.8 (81.0)	7.0 (82.1)
Overall *B* factor from Wilson plot (Å^2^)	43	63
