# Supplementary material for: Crystallization and X-ray diffraction analysis of an l-arabinonate dehydratase from Rhizobium leguminosarum bv. trifolii and a d-xylonate dehydratase from Caulobacter crescentus
Source: Acta Crystallogr F Struct Biol Commun. 2016 Jul 13;72(Pt 8):604–8. doi: 10.1107/S2053230X16010311 (PMC4973301; doi:10.1107/S2053230X16010311)
Supplement: Supplementary file 1 [file f-72-00604-sup1.pdf]

Acta Cryst F

STRUCTURAL BIOLOGY
COMMUNICATIONS

Volume 72 (2016)

Supporting information for article:

Crystallization and X-ray diffraction analysis of an L-arabinonate dehydratase from *Rhizobium leguminosarum* bv. *trifolii* and a D-xylonate dehydratase from *Caulobacter crescentus*

Mohammad Mubinur Rahman, Martina Andberg, Anu Koivula, Juha Rouvinen and Nina Hakulinen

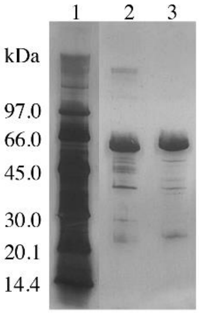

Figure S1 SDS-gel after silver staining. Lane 1 contains low-molecular-weight protein standards, lane 2 *Rl*ArDHT and lane 3 *Cc*XyDHT.
